# Gamma distribution based predicting model for breast cancer drug response based on multi-layer feature selection

**DOI:** 10.3389/fgene.2023.1095976

**Published:** 2023-02-02

**Authors:** Tongtong Cui, Zeyuan Wang, Hong Gu, Pan Qin, Jia Wang

**Affiliations:** ^1^ Faculty of Electronic Information and Electrical Engineering, Dalian University of Technology, Dalian, Liaoning, China; ^2^ Department of Breast Surgery, Second Hospital of Dalian Medical University, Dalian, Liaoning, China

**Keywords:** drug response, machine learning, feature selection, breast cancer, generalized linear model, artificial neural network

## Abstract

In the pursuit of precision medicine for cancer, a promising step is to predict drug response based on data mining, which can provide clinical decision support for cancer patients. Although some machine learning methods for predicting drug response from genomic data already exist, most of them focus on point prediction, which cannot reveal the distribution of predicted results. In this paper, we propose a three-layer feature selection combined with a gamma distribution based GLM and a two-layer feature selection combined with an ANN. The two regression methods are applied to the Encyclopedia of Cancer Cell Lines (CCLE) and the Cancer Drug Sensitivity Genomics (GDSC) datasets. Using ten-fold cross-validation, our methods achieve higher accuracy on anticancer drug response prediction compared to existing methods, with an *R* ^2^ and RMSE of 0.87 and 0.53, respectively. Through data validation, the significance of assessing the reliability of predictions by predicting confidence intervals and its role in personalized medicine are illustrated. The correlation analysis of the genes selected from the three layers of features also shows the effectiveness of our proposed methods.

## 1 Introduction

Due to the advances in technology, medical devices and treatment conditions, cancer is no longer as difficult to treat as it was 20 years ago. However, the heterogeneity and genetic diversity of cancer make it possible for patients with the same type of cancer to obtain different therapeutic effects even with the same treatment (Stein et al., 2004; Shoemaker, 2006; Workman et al., 2012; Cortés-Ciriano et al., 2015). Although the research on drug sensitivity is widespread and many methods have already demonstrated outstanding performance in this research field, it is still challenging to develop more accurate and powerful computational models to improve the performance of prediction. Moreover, portable algorithm development is a hot topic in this area (Caponigro and Sellers, 2011; Garnett et al., 2012; Wei et al., 2019).

At present, precision medicine is a crucial issue in cancer treatment research around the world (Reardon, 2015; Ali Dokuyucu et al., 2018; Li and Li, 2022), which needs to take into account patient information such as medical history and genetic information, resulting in individualized treatment plans for patients with maximum therapeutic effects and minimum side effects. Since cancer is a disease caused by genetic mutations, it is reasonable to develop computational models based on the genetic data of patients to predict drug responses (Garraway, 2013). Based on the data on cancer patients accumulated over the past few decades, several large public cancer datasets have emerged. The Encyclopedia of Cancer Cell Lines (CCLE) project compiled the genomic profiles of 947 human cancer cell lines and the pharmacological profiles of 24 anticancer drugs in 479 cancer cell lines (Barretina et al., 2012). The Cancer Drug Sensitivity Genomics (GDSC) is another project which compiled the genomic maps of 639 human cancer cell lines and their drug response data into 130 drugs, aiming to identify genomic biomarkers of drug sensitivity in cancer cells (Yang et al., 2013). Both CCLE and GDSC datasets have abundant genomic data, including gene expression, DNA copy number, ONcomAP mutation, *etc.*, which offer support for the construction of prediction models.

Machine learning methods, such as Random Forest (RF) and the Bayesian approach, are commonly used to establish fitting and regression models of drug response prediction models. Fang et al. used the EC50 of 947 cancer cell lines in the CCLE database to predict drug response based on RF, and then estimated the conditional distribution by observing the weight distribution of tag values (Fang et al., 2018). Finally, they obtained the point estimate of drug response value, and established the prediction interval to evaluate the prediction credibility. Amid-ud-din et al. proposed a Kernelized Bayesian Matrix Factorization (KBMF) based algorithm, which integrates genomic features of cell lines such as gene expression data, copy number variation and gene mutations as auxiliary information for predicting the drug response of 650 cell lines to 116 drugs, achieving an *R* ^2^ of 0.32 for the new drugs prediction (Ammad-ud din et al., 2014). The methods based on fuzzy-rough set evaluation have also been applied to the feature selection problem, where lower and upper approaches are used to intuitive fuzzy sets from rough sets to remove uncertainty due to having simultaneous membership, non-membership, and hesitation degrees and obtain better results (Lanbaran and Celik, 2021; Lanbaran et al., 2022). In addition, there have been some attempts based on deep learning. Menden et al. made the first effort to integrate cell line genomic features, including microsatellites, sequence variation and copy number variation, combined with one-dimensional (1D) and two-dimensional (2D) chemistry of compounds to model half growth inhibitory concentration (IC50) (Menden et al., 2013). The IC50 of 111 drugs were predicted on 608 cell lines using three-layer neural networks and random forest. As a result, the coefficient of determination (*R* ^2^) and the root mean square error (RMSE) are 0.64 and 0.97 on the test set, respectively.

In addition to the improvement of the algorithm itself, the optimization of the input data is another effort which have been made to improve the performance of the model. Cortes-Ciriano et al. (2016) compared seven genomic profiles and their cell line combinations and found that protein, gene transcript levels and miRNA abundance had the highest predictive power when simulating the 50% growth inhibition bioassay endpoint. They then integrated the transcriptional profiles of the top 1,000 genes that showed the highest variance in 59 cell lines, as well as the Morgan fingerprints of 17,142 compounds, and used RF and support vector machines (SVM) to predict drug response. Zhang et al. (2015) constructed a three-layer integrated cell line drug network including cell line similarity network (CSN) and drug similarity network (DSN) based on Pearson’s correlation coefficient of cell line gene expression profile and compound 1D and 2D information from CCLE and the Cancer Genome Project (CGP) datasets. The basic assumption is that similar drugs may have similar responses to a given cell line. In the proposed model, the drug response is first inferred from each network, and then the final response is obtained by linear weighting, with weights customized for each drug. The Pearson’s correlation coefficient between the predicted drug response and the observed response is 0.6. However, there are still some problems in the existing studies. For example, the results of most studies are obtained by statistical analysis and have not been verified in new cell lines. From the perspective of data sources, although the genomic information such as methylation, copy number variation, and gene mutation are considered in several previous studies, other information such as drug-target interaction is not included.

**FIGURE 1:**
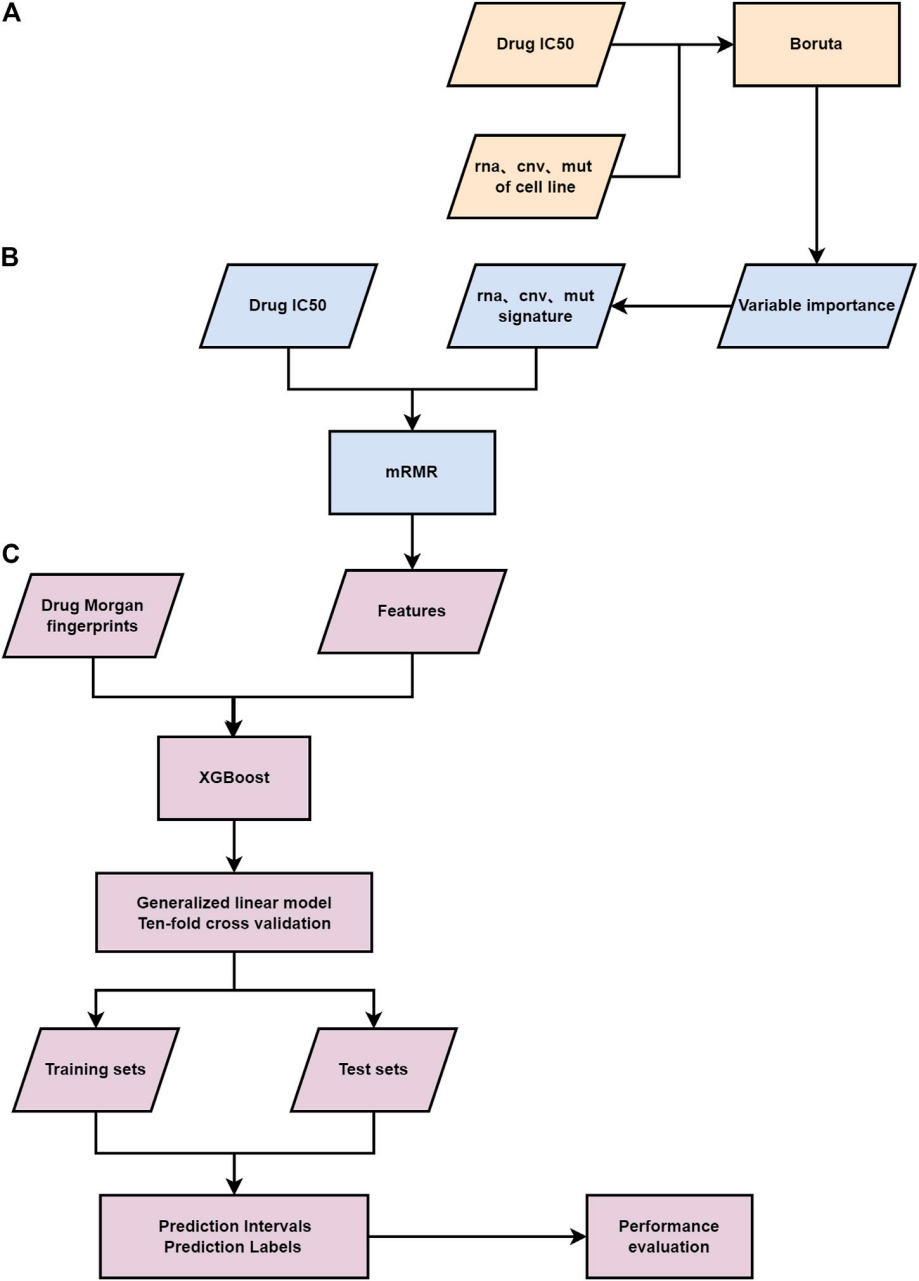
Flowchart of the study. **(A)** The first-layer feature selection is performed on the genomic information of patients. **(B)** The second feature selection layer. **(C)** The third layer feature selection is carried out for the Morgan fingerprints and genomic features of drugs, a gamma distribution based GLM and ten-fold cross-validation are applied to the final feature matrix.

To further improve the prediction accuracy for drug response, we propose a three-layer feature selection combined with a gamma distribution based generalized linear model (GLM), the flowchart of which is shown in Figure 1 and a two-layer feature selection combined with an artificial neural network (ANN) for drug response prediction. Three feature selecting methods, namely Boruta (Xu et al., 2019), mRMR (Junhuai et al., 2016) and XGBoost (Sidorov et al., 2018) are applied on the drug Morgan molecular fingerprint coding and genomic data. After the feature selection, the gamma distribution based GLM and ANN are applied to the feature matrix to predict specific IC50 value (Cheng et al., 2016) of the drug for cancer cell lines. In general, our proposed models outperform the existing models such as RF and the Bayesian model, while predicting precise confidence intervals for the IC50 values for breast cancer, which can select appropriate drugs for cancer treatments.

## 2 Methods

### 2.1 Data and preprocessing

In this study, the gene expression and drug IC50 data from CCLE and GDSC databases are used. We focus on four classes of drugs for breast cancer: Anthracycline, Paclitaxel, Cyclophosphamide and Platinum. Since there are two types of Anthracycline, a total of five drugs are studied. The gene expression data are used as features, including gene mutations (MUT), chromosomal variations (RNA), and copy number variations (CNV). Among them, the chromosomal and copy number variations are real numbers, while gene mutations are binary, with 1 representing mutation and 0 representing wild-type.

In addition, the 2D chemical structures of the five drugs are downloaded from the PubChem website, whose Morgan fingerprints are calculated using the R package RCDK (Guha, 2007). The Morgan fingerprint is a topological fingerprint, which is obtained by the modified Morgan algorithm (Duvenaud et al., 2015). The algorithm first assigns a unique identifier to each atom. Then, after iterations of updating, the substructure is calculated, generating a 256-bit binary feature list.

We use two types of feature matrices as input. One is composed of the gene expression data alone, for which the label values keep intact, and regression fitting is carried out for the five drugs respectively. The other feature matrix is integrated with the gene expression data and Morgan fingerprints, for which all cell lines from the five drugs are integrated and the label values are logarithmically transformed.

### 2.2 Feature selection

Since the dimension of feature matrix of gene expression data reaches tens of thousands, it is necessary to use feature selection to get key features to reduce the size of the model. In this study, a three-layer feature selection integrating Boruta (Xu et al., 2019), mRMR (Junhuai et al., 2016) and XGBoost (Sidorov et al., 2018) is adopted. We decide to use a three-layer feature selection for three reasons. Firstly, we aim to select all the feature sets related to the label value, rather than selecting the feature set that can minimize the loss function for a specific model. Secondly, for ultra-high-dimensional features, the program of the regression model may crash after single or double-layer feature selections. Thirdly, the three-layer feature selection algorithm can help understand the influencing factors of label values more comprehensively, so as to perform feature selection better and more efficiently.

#### 2.2.1 Boruta

A preliminary screening of the features is first applied by training two single-hidden-layer autoencoder networks, where the hyperbolic tangent is used as the activation function. The contribution of input genes to output genes is calculated to screen the chromosomal variation and copy number variation features. Based on the Gedeon method, the contribution *Q* of the *i*th input gene to the *j*th output gene is expressed as

$$Q_{ij}=\overset{K}{\underset{k=1}{\sum }}\left(P_{ik}\times P_{kj}\right)$$ (1)

where K denotes the total number of the neurons of the hidden layer. *P*_*ik*_ is the contribution of the *i*th input to the *k*th neuron of the hidden layer calculated by

$$P_{ik}=\frac{\left|W_{ik}\right|}{\overset{G}{\underset{i^{*}=1}{\sum }}\left|W_{i^{*}k}\right|}$$ (2)

with *G* being the total number of the inputs and $W_{i^{*}k}$ being the weight linking the corresponding neuron couple. *P*_*kj*_ is the contribution of the *k*th neuron of the hidden layer to the *j*th output, whose calculation is similar to that of *P*_*ik*_. The total contribution of the *i*th input is calculated by

$$q_{i}=\overset{G}{\underset{j=1}{\sum }}\frac{Q_{ij}}{\overset{G}{\underset{i^{*}=1}{\sum }}Q_{i^{*}j}}$$ (3)

We rank the inputs of the autoencoder in descending order with respect to *q*_*i*_ and remain the last 50% features. Then, we retain one feature from the highly correlated features, whose correlation coefficients are more than 0.8. The extracted rna and cnv features are finally merged with the mut features, resulting in a matrix with about 23,500 dimensions. Compared with the original feature matrix, the size is reduced by more than half. However, such magnitude can still lead to the curse of dimensionality. To avoid such problem, the mRMR algorithm is carried out to further reduce the number of features.

#### 2.2.2 mRMR

An ideal list of features should have two properties: a strong correlation with the object variable, and no redundancy among features. Based on this criterion, we apply the mRMR algorithm which selects features by calculating the mutual information between features and the object variable. The mutual information entropy between feature *X* and the response variable (class label) *Y* can be calculated as follows:

$$I\left(Y,X\right)=\int_{\Omega_{Y}}\int_{\Omega_{X}}p\left(x,y\right)\log\left(\frac{p\left(x,y\right)}{p\left(x\right)p\left(y\right)}\right)dxdy$$ (4)

where Ω_*Y*_ and Ω_*X*_ are the sample spaces corresponding to *Y* and *X*, *p*(*x*, *y*) is the joint probability density, and *p*() is the marginal density function. Assuming there are in total *m* features, and for a given feature *X*_*i*_(*i* ∈ {1, 2, … , *m*}), its feature importance based on the mRMR criterion can be expressed as:

$$f^{mRMR}\left(X_{i}\right)=I\left(Y,X_{i}\right)-\frac{1}{\left|S\right|}\underset{X_{s}\in S}{\sum }I\left(X_{s},X_{i}\right)$$ (5)

where *S* is the set of selected features, |*S*| is the size of the feature set (number of features), *X*_*s*_ ∈ *S* is one feature out of the feature set *S*, *X*_*i*_ denotes a feature currently not selected: *X*_*i*_∉*S*.

In the mRMR feature selection process, at each step, the feature with the highest feature importance score $\max_{X_{i}\notin S}f^{mRMR}\left(X_{i}\right)$ will be added to the selected feature set *S*. By setting *m*, a total of 500 features are finally selected.

#### 2.2.3 XGBoost

When integrating the Morgan fingerprints of drugs into the feature matrix, it is inevitable to generate a number of missing values, which may cause the model to fail. To deal with this problem, we apply XGBoost which can automatically learn the splitting direction for samples with missing data, while reducing the feature dimension. Based on the modification to the (Gradient boosted decision tree, GBDT) model which uses the first derivative, the XGBoost algorithm makes a second-order Taylor expansion of the loss function, while adding a regularization term to the objective function, which is used to balance the complexity of the objective function and the model to prevent overfitting. The objective function is expressed as:

$$\Psi_{m}=\overset{N}{\underset{i=1}{\sum }}\left[g_{i}f_{m}\left(x_{i}\right)+\frac{1}{2}h_{i}f_{m}^{2}\left(x_{i}\right)\right]+\Omega \left(f_{m}\right)$$ (6)

where ${\left\{\left(x_{i},y_{i}\right)\right\}}_{i=1}^{N}$ is the training set, *f*_*m*_ represents the spanning tree model in the *m*th iteration. Let $F_{m}\left(\right)$ be the prediction at the *m*th iteration, we represent the first and second order gradient statistics on the loss function as $g_{i}=\frac{\partial \Psi \left(y_{i},F_{m-1}\left(x_{i}\right)\right)}{\partial F_{m-1}\left(x_{i}\right)}$ and $h_{i}=\frac{\partial^{2}\Psi \left(y_{i},F_{m-1}\left(x_{i}\right)\right)}{\partial F_{m-1}{\left(x_{i}\right)}^{2}}$ , respectively. For the regularization part, both L1 and L2 regularizations, as well as other approaches such as adding weights to the boosting of the tree at each step and sampling feature columns have been tested. The L2 regularization $\Omega \left(f_{m}\right)=\gamma L_{m}+\frac{1}{2}\lambda {\left\|\omega_{m}\right\|}_{2}^{2}$ is finally chosen to penalize the complexity of the model, where *L*_*m*_ represents the number of leaf nodes of the spanning tree model *f*_*m*_, $\omega_{m}=\left(\omega_{m1},\omega_{m2},\ldots ,\omega_{m_{L_{m}}}\right)$ represents the output value of each leaf node of *f*_*m*_. *γ* and *λ* are the regularization coefficients.

**FIGURE 2:**
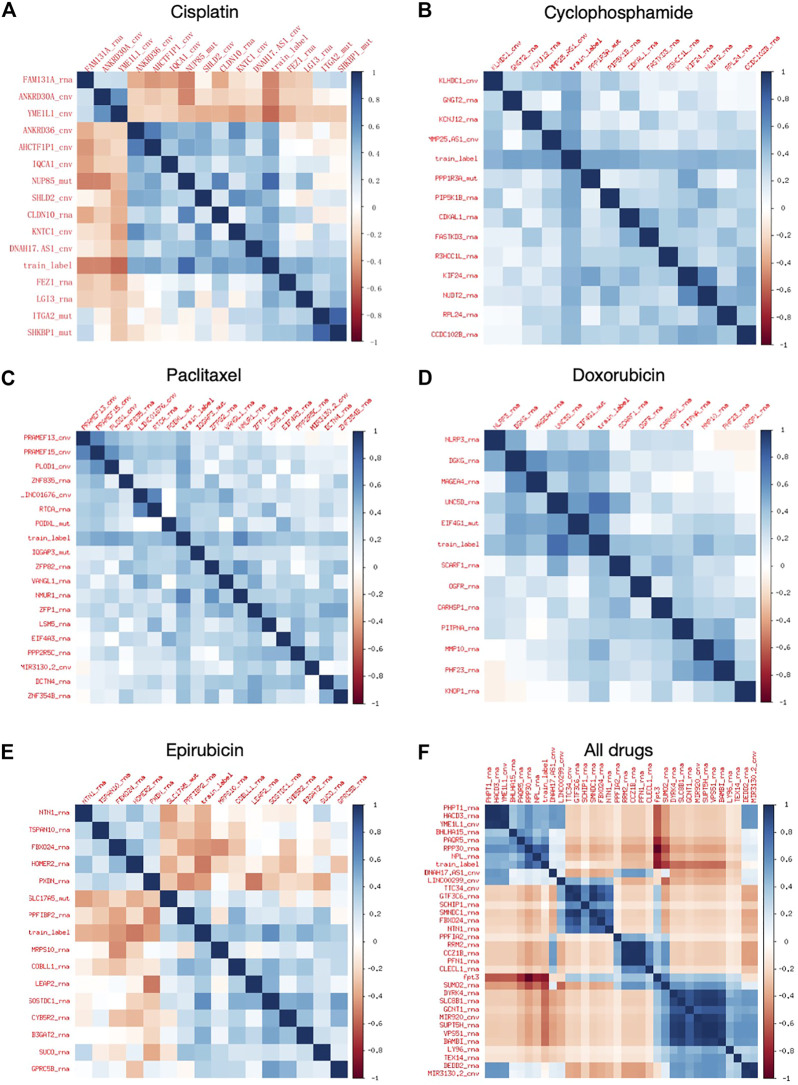
Heat map of correlation coefficients of extracted features. **(A–E)** Feature heat maps for Cisplatin, Cyclophosphamide, Paclitaxel, Doxorubicin, and Epirubicin, respectively. **(F)** Heat map of combined feature extraction for all five drugs.

After implementing XGBoost, the feature dimension is reduced to below 40. The number of features of each drug selected by each layer is shown in Table 1. The heat maps of the correlation coefficients of the feature matrices for the analyzed drugs are shown in Figure 2.

**TABLE 1:** Number of features selected by three-layer feature selection.

Drug	Initial dimension	Boruta	mRMR	XGBoost
Epirubicin	41× 49,149	41× 23,356	41× 500	41× 29
Cisplatin	46× 49,149	46× 23,551	46× 500	46× 28
Cyclophosphamide	42× 49,149	42× 23,353	42× 500	41× 33
Doxorubicin	45× 49,149	45× 23,476	45× 500	45× 32
Paclitaxel	46× 49,149	46× 23,531	46× 500	46× 27
All drugs	—	—	220× 2,370	220× 30

The number of features retained after each step of feature selections are included in Supplementary Material S1, and the specific features are listed in Supplementary Material S1. It can be seen from Supplementary Table S3 that the above table that Boruta mainly screens the features of the RNA and CNV types, and retains most of the features of the MUT type. mRMR further screens all three types of features, with RNA and MUT types being more important. XGBoost retains feature types dependent on specific drugs. Overall, for the regression models, the RNA features contribute the most.

### 2.3 Regression models for drug response prediction

Regression has been an important procedure to predict drug response. In this study, two different machine learning methods are selected namely gamma distribution based GLM and ANN, which are particularly effective for data with non-linear and non-constant variance structures. A GLM consists of three elements: a particular distribution for modeling *Y* from among those which are considered exponential families of probability distributions, a linear predictor *η* = *Xβ*, and a link function *g* such that *E*(*Y*∣*X*) = *μ* = *g* ^−1^(*η*). Since the drug IC50 values are positive, we tested exponential distribution, gamma distribution, and inverse Gaussian distribution on the reduced set of features. To avoid overfitting, the optimal regression model is chosen according to the Akaike information criterion (AIC), which can find the best balance between model complexity and likelihood function. With the minimal AIC among all the candidate models, the GLM with the gamma distribution is finally chosen, whose probability density function is as follows:

$$f\left(x\right)=\frac{1}{\Gamma \left(k\right)\theta^{k}}x^{k-1}e^{-\frac{x}{\theta }}$$ (7)

where *k* is the shape parameter, *θ* is the scale parameter. *γ*(*k*) is the gamma function with the following form:

$$\Gamma \left(k\right)=\int_{0}^{\infty }\mu^{k-1}e^{-\mu }d\mu$$ (8)

**FIGURE 3:**
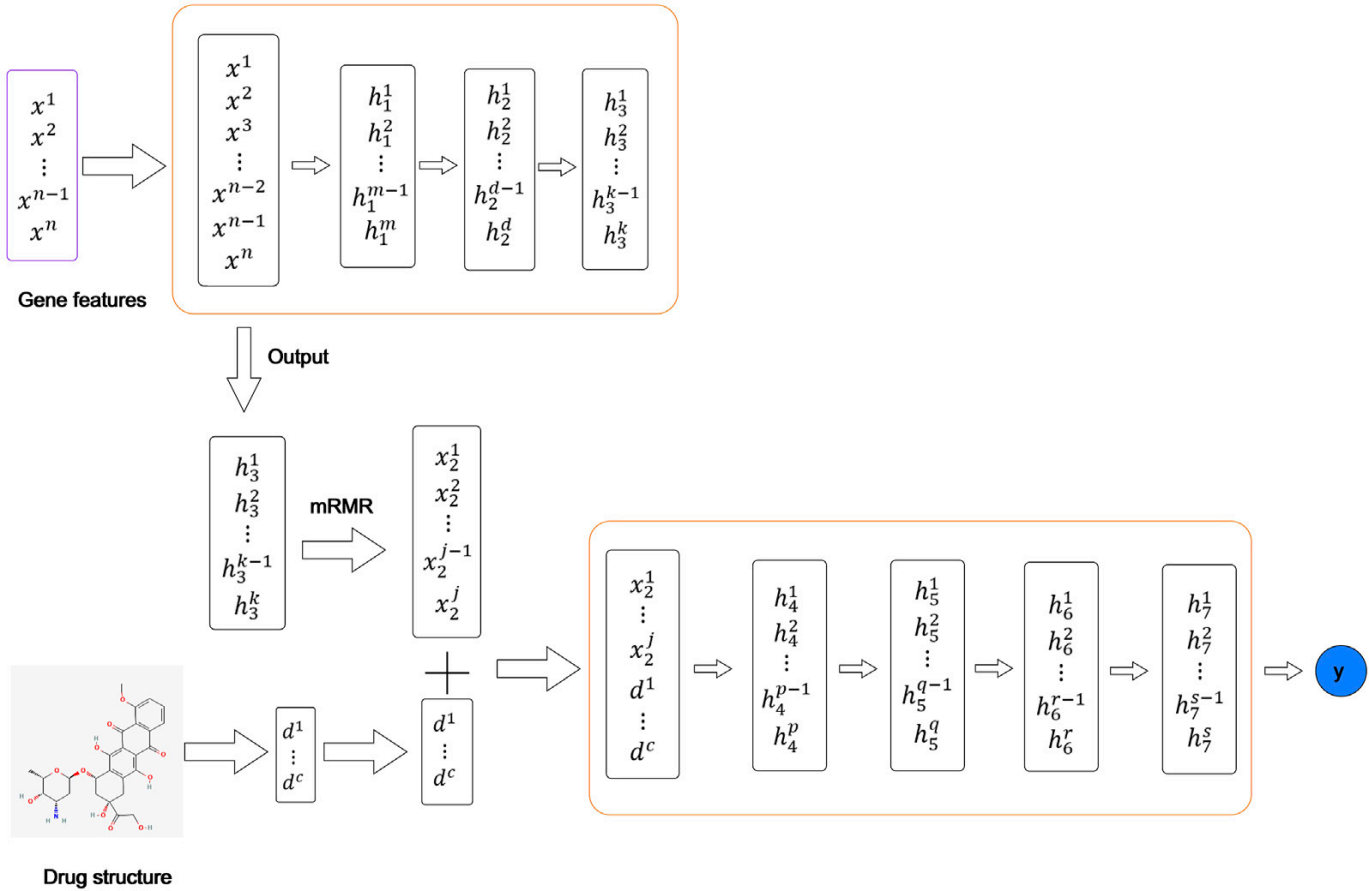
ANN structure for drug response prediction.

ANN is a hierarchical feature learning approach, which has gained attention in recent years mainly because of its solid performance for supervised learning. Due to its ability to extract features from data through a series of hidden layers with non-linear transformations, the first two layers of pre-feature selection are adopted. The ANN is implemented in R using the H2O package (LeDell et al., 2018). For the number of hidden layers of ANN, we have tried from three layers to six layers. For the number of nodes, multiple combinations from 100–2000 have been tried. According to the *R* ^2^ and RMSE values, the optimal ANN includes four hidden layers with 1,000, 800, 500, 100 notes, respectively. A regularization term is added in order to prevent overfitting. The activation function is TanhWithDropout, and RMSE is used as the loss function. The ANN framework is shown in Figure 3.

## 3 Results

In order to assess the ability of the proposed framework to predict drug response, ten-fold cross-validation is applied to the trained gamma distribution based GLM and ANN. For each model based on five drugs, three evaluation metrics are used, namely root mean square error (RMSE), coefficient of determination (*R* ^2^) and prediction interval coverage probability (PICP). Among them, RMSE is more sensitive to errors and is more suitable for measuring the quality of drug sensitivity models. *R* ^2^ is also a common statistic reflecting model fit. PICP is an evaluation criterion for interval estimation, which is used to assess the confidence interval for drug response. Table 2 shows the evaluation criteria of different methods for different drugs under different input data types. Using gene expression data and drug structure as input, our model performs well on all criteria.

**TABLE 2:** Model performance evaluation. The bold values indicate the better results of the two approaches for each drug.

Input	Approach	Drug	*R* ^2^	RMSE	PICP
Gene expression	GLM	Epirubicin	0.823	1.12	0.81
		Cisplatin	**0.947**	**0.36**	1
		Cyclophosphamide	**0.884**	**0.32**	1
		Doxorubicin	**0.849**	**1.43**	0.87
		Paclitaxel	0.745	**1.61**	0.77
	ANN	Epirubicin	**0.83**	**0.95**	1
		Cisplatin	0.938	2.55	0.75
		Cyclophosphamide	0.811	1.88	0.74
		Doxorubicin	0.842	1.62	0.84
		Paclitaxel	**0.948**	1.97	0.76
Gene expression+Morgan fingerprint	GLMANN	All drugsAll drugs	**0.874**0.36	**0.57**1.33	0.910.99

**FIGURE 4:**
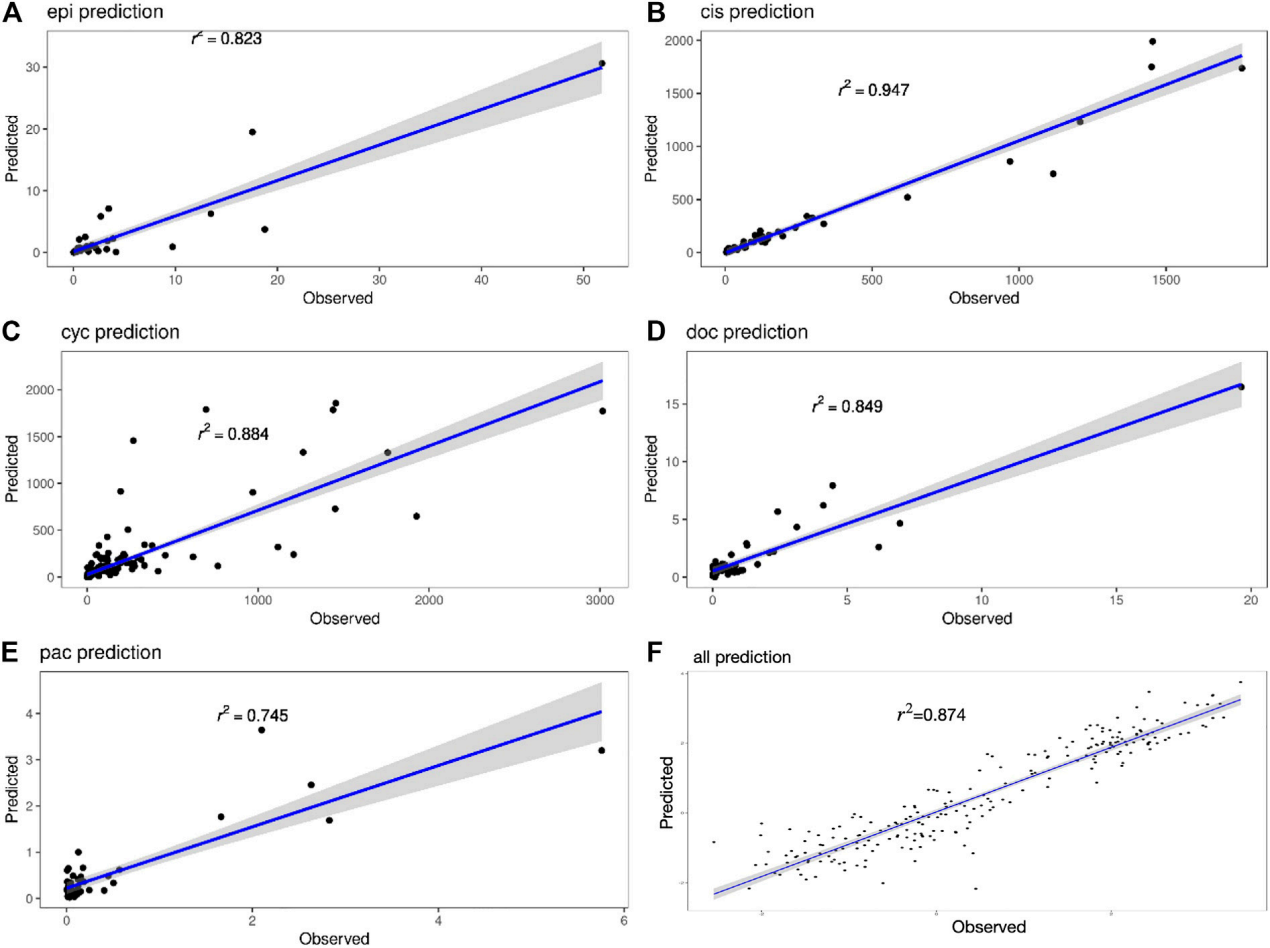
**(A–E)** Scatter plots of true and predicted values of Epirubicin, Cisplatin, Cyclophosphamide, Doxorubicin and Paclitaxel. **(F)** Scatter plot of true and predicted values for all 220 samples.

Figure 4 shows the true value-predicted value scatter plots of three-layer feature selection-GLM. Among the five drugs, the average *R* ^2^ is 0.849, with Cisplatin reaching the highest *R* ^2^ value of 0.947, which is slightly better than the ANN model, indicating that the proposed models reveal solid performance for drug sensitivity prediction.

**FIGURE 5:**
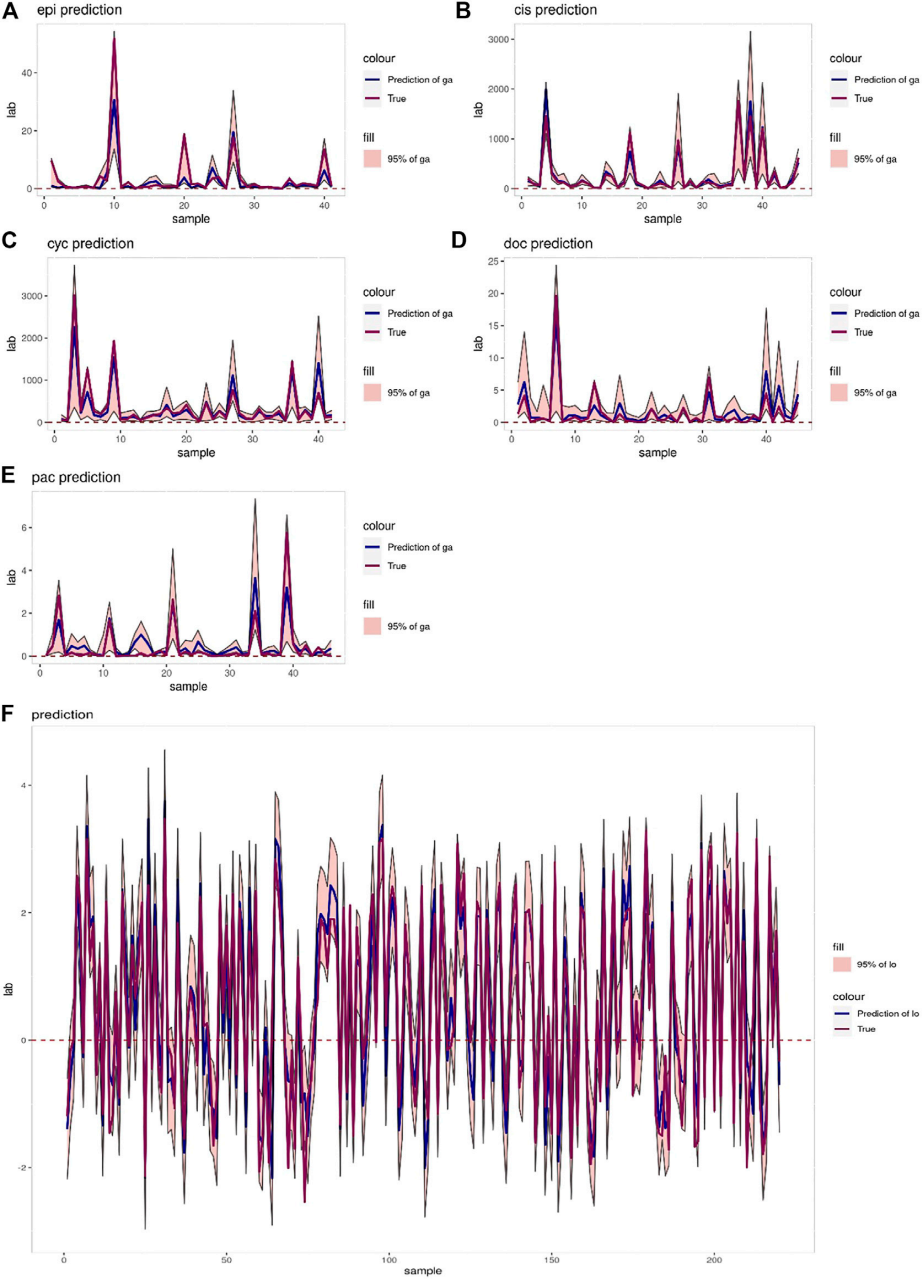
**(A–E)** Prediction intervals (blue lines represent predicted values, red lines represent true values, pink areas represent prediction intervals with 95% confidence level) of Epirubicin, Cisplatin, Cyclophosphamide, Doxorubicin and Paclitaxel. **(F)** Interval prediction results for all 220 samples.

The interval prediction results for five drugs are shown in Figure 5 indicating that the predicted and true values of the drugs generally fall within the prediction interval calculated by the model (see Table 2 for the specific prediction interval coverage). In addition, Figure 4F and Figure 5F indicate that the model performance does not deteriorate after combining the inputs, with an *R* ^2^ value of 0.874. The advantage of adding molecular Morgan fingerprint is that after a new drug is invented, the Morgan molecular fingerprint can be calculated to obtain a similarity matrix with existing drugs, then the IC50 value can be calculated by using the prediction model, which provides new options for the development and performance testing of new drugs.

To further illustrate the effectiveness of the three-layer feature selection-generalized linear regression model, we compare three existing algorithms, including RF-XGBoost (Fang et al., 2018), ANN-RF (Menden et al., 2013) and RF-SVM (Hejase and Chan, 2015), as shown in Table 3. Among them, RF-XGBoost is primarily modeled on drug synergy data to predict synergy scores of each cell line. ANN-RF method takes genomic features and medicinal chemical descriptors (including physicochemical features such as body weight, lipophilicity and fingerprints) as input, and the neural network can impute missing values, using eight-fold cross-validation to obtain R-square and RMSE. The RF-SVM method sets the trees in the forest to 100, using mean square error as the criterion for evaluating the split quality, and then uses ten-fold cross-validation and grid search to optimize the parameters.

**TABLE 3:** Model performance comparison. The bold values indicate the best results of the models.

Model	Input data type	*R* ^2^	RMSE
XGBoost + RF	Drug synergy data + medicinal chemical characterization	0.74	—
ANN + RF	Genomic features + drug smiles fingerprints	0.64	0.97
RF + SVM	Genome features + drug compound structure	0.78	0.52
Three-layer feature selection + GLM	Genomic features + drug Morgan fingerprints	**0.87**	**0.51**

Beyond that, we have done a series of extra experiments, including a cross experiment to test the reliability of the model when facing new datasets, as well as predicting with one and two layers of feature selections. The results are included in Supplementary Material S1. According to the Supplementary Figure S1; Supplementary Table S1, although the performances of our proposed models slightly degrade due to the inconsistency between databases, the models are still reliable when applied to new data. It can be seen from Supplementary Figure S2, S3; Supplementary Table S2 that the performances of the regression models using single or double-layer feature selections are unsatisfactory, indicating that using multiple feature selections based on different principles can better eliminate redundancy and screen out key features.

## 4 Discussion

**FIGURE 6:**
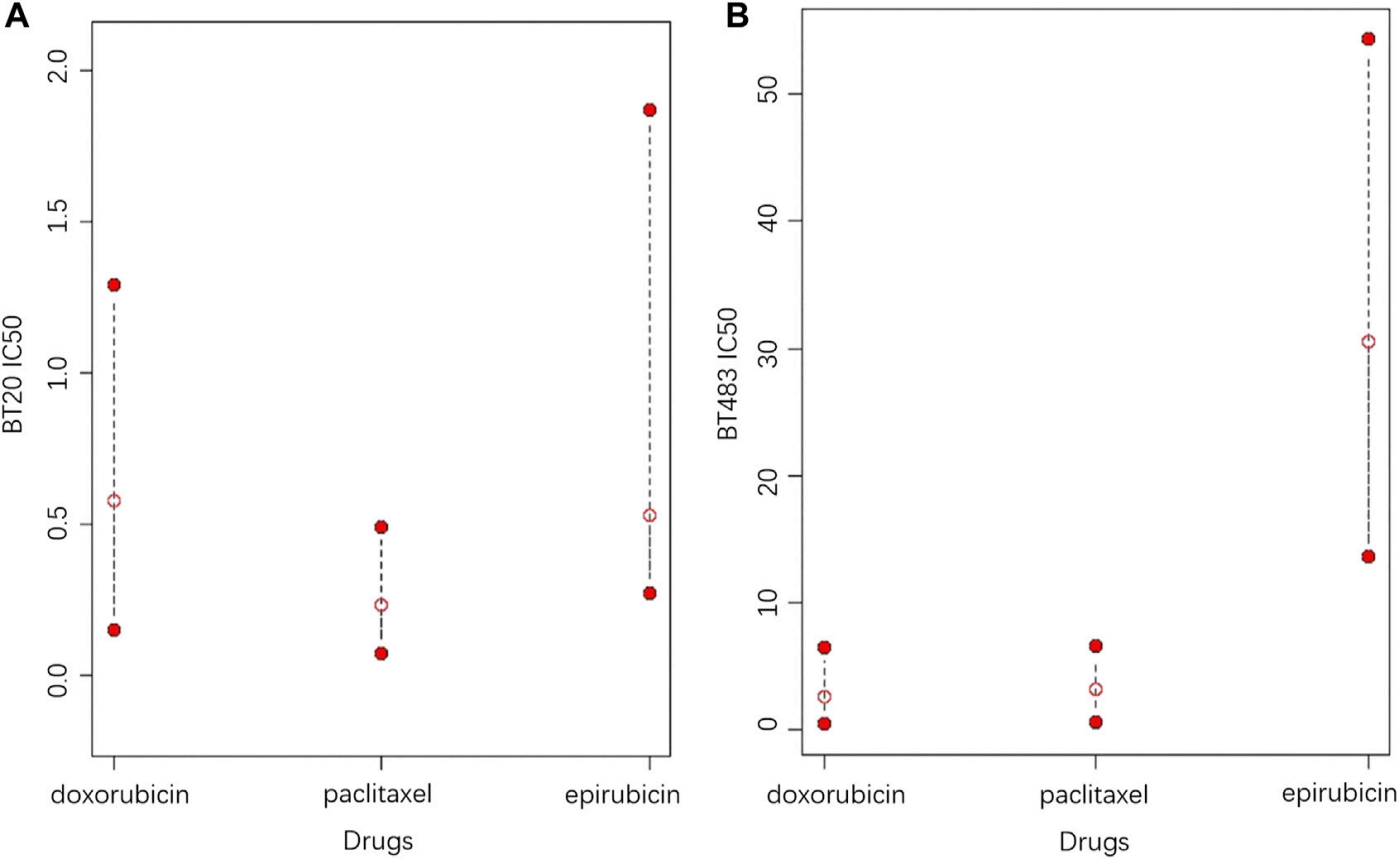
The 95% prediction intervals and point predictions of Epirubicin, Doxorubicin and Paclitaxel for cell lines **(A)** BT20 and **(B)** BT483 (the open red dots represent the point prediction of drug response, and the two solid red dots represent the upper and lower bounds of the 95% prediction interval).

In contrast to the commonly used point predictions, confidence intervals can give a range that includes a high probability of drug response and assess reliability by the interval length. At a given confidence level, a shorter confidence interval indicates less fluctuation in the drug response, meaning that the efficacy of the drug is more reliable. Although the assessment of drug efficacy based on the length of the confidence interval is relatively intuitive, it is not statistically rigorous, thus the homogeneity test of drug response variance is applied to provide more reliable statistical proofs. To better explain, an example of the CCLE dataset is given, in which the potential treatment options for two cell lines are explored among Epirubicin, Doxorubicin and Paclitaxel, as shown in Figure 6. It can be seen in Figure 6A that Doxorubicin and Paclitaxel show little difference in point prediction. However, in terms of confidence interval prediction, Paclitaxel shows a shorter interval compared to Doxorubicin, and the *p*-value for the homogeneity of variance test for the two drugs is 4.996 × 10^−8^. In addition, the true IC50 values of Doxorubicin and Paclitaxel for this sample are 1.03 and 0.024, respectively. Therefore, Paclitaxel is optimal for treatment, which is more effective and stable.

In the second case, according to Figure 6B, Doxorubicin is the best choice based on the point prediction results. However Paclitaxel, which has the second lowest point predicted value, gives a shorter prediction interval than Doxorubicin. Therefore, considering the stability of the treatment effect, Paclitaxel should be a better choice. Furthermore, in both cases, Epirubicin has higher upper and lower predictive bounds compared to Doxorubicin and paclitaxel, meaning that Epirubicin is a more aggressive option with higher risks. In general, Paclitaxel is suitable for conservative treatment, while choosing Doxorubicin or Epirubicin take more risks. Based on the analysis of this example, it can be concluded that the confidence intervals provide more information for drug response prediction, meanwhile providing more sensible recommendations for treatments.

## 5 Conclusion

In this paper, a three-layer feature selection-GLM and a two-layer feature selection-ANN are proposed to give point and confidence interval predictions of drug responses, which are based on the genomic features as well as the chemical structure of drugs. The results indicate that the proposed models reveal solid performance for drug sensitivity prediction. In order to evaluate the difference between the two prediction intervals, we also propose a homogeneity test of the variance between patients, and illustrate the reliability of the prediction confidence interval through the homogeneity test. We hold the opinion that this study makes a valuable contribution to the field in three aspects. First and foremost, as predicting models for drug response, the practicality of the models has been proved by experiments. Secondly, the proposed models help realize precision medicine by not only predicting the point values, but also calculating the confidence intervals of drug responses, which provide additional information for treatment selections. Thirdly, in the promissing field of drug repositioning, which explores new indications of drugs by using existing drugs or drugs with failed clinical trials, machine learning methods have obvious advantages in terms of time and cost (Yang et al., 2022). Our proposed models are able to provide strong support by giving reliable predictions of drug responses.

Admittedly, there are still some deficiencies for future research. First of all, although the neural network we used reveals decent accuracy, it loses the interpretability of the features, which may be the cause of the high RMSE values. Thus, finding interpretable predictors for drug response will be our future goal. In addition, in this work, we mainly compare the reliability of drug response prediction intervals through statistical inference, while lacking corroboration of clinical experiments. In the future, with the support of clinical medical data, the completeness and credibility of our research can be increased.

## Data Availability

The original contributions presented in the study are included in the article/Supplementary Material, further inquiries can be directed to the corresponding authors.
