# Supplementary material for: Gamma distribution based predicting model for breast cancer drug response based on multi-layer feature selection
Source: Front Genet. 2023 Feb 2;14:1095976. doi: 10.3389/fgene.2023.1095976 (PMC9932661; doi:10.3389/fgene.2023.1095976)
Supplement: Supplementary file 1 [file DataSheet1.PDF]

Supplementary Material

1 FIGURES

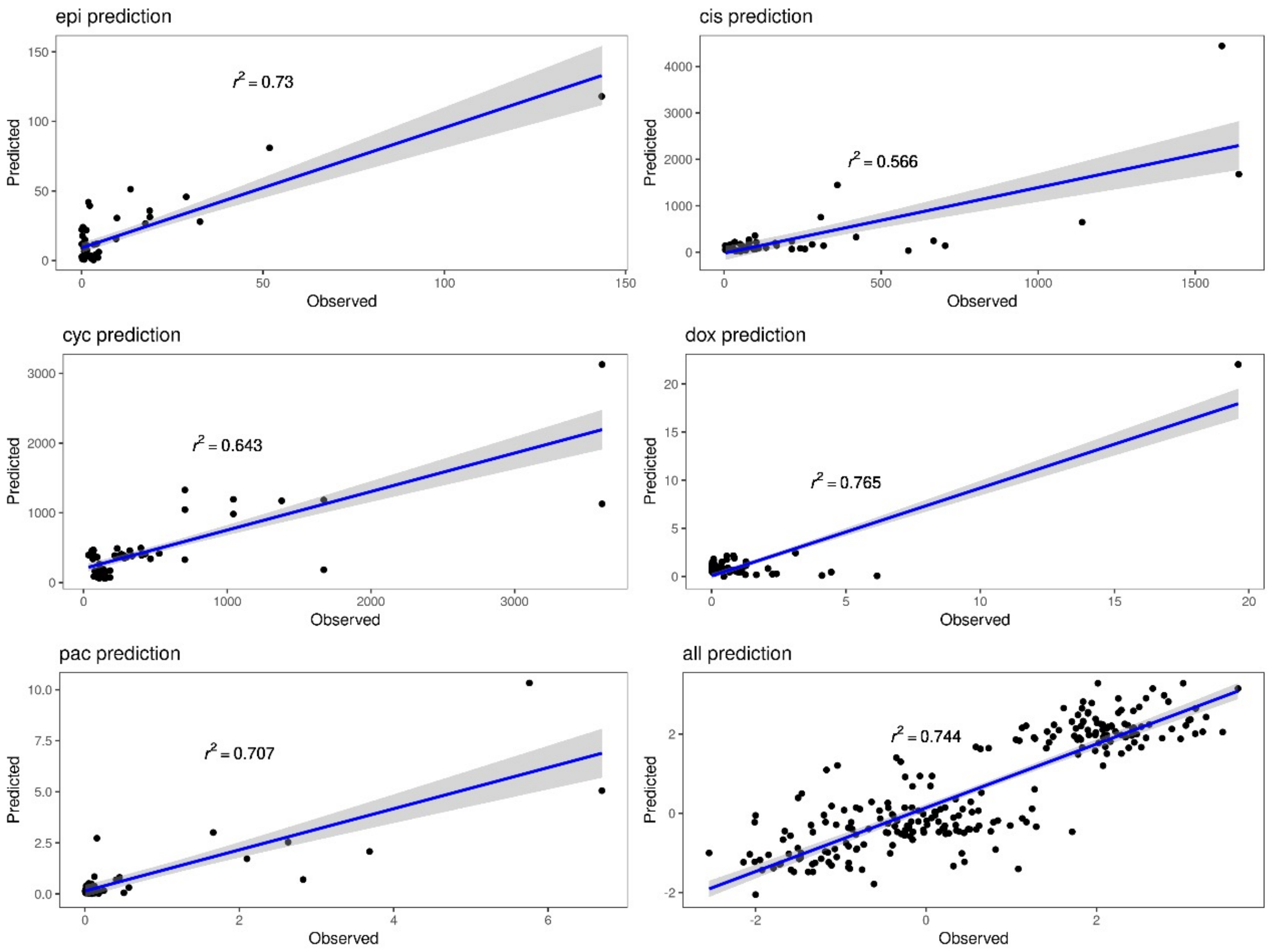

Figure S1. Scatter plots of true and predicted values of five drugs and their combination. The three-layer feature selection-GLM is trained on CCLE and tested on GDSC.

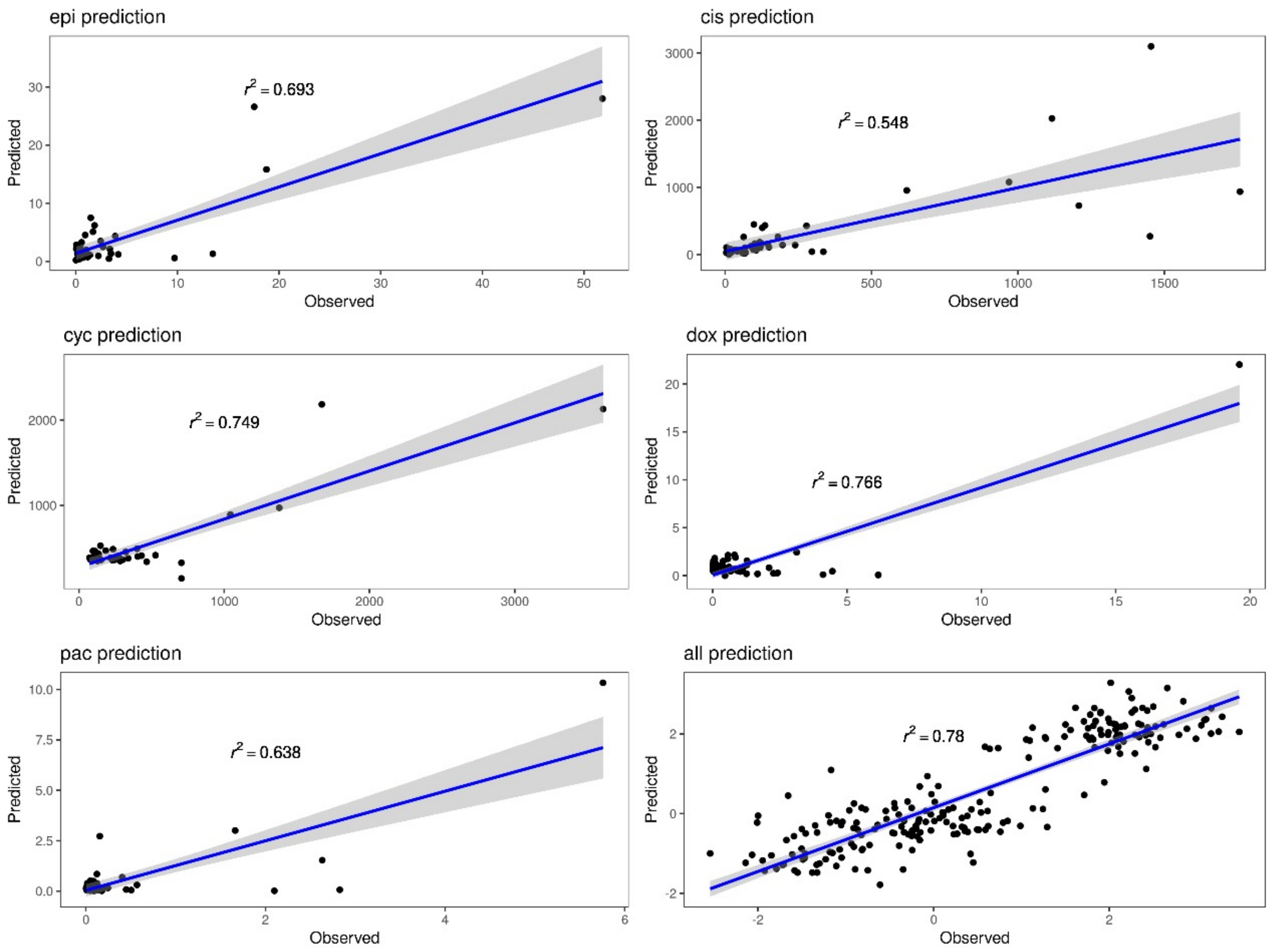

Figure S2. Scatter plots of true and predicted values of five drugs and their combination. The three-layer feature selection-GLM is trained on GDSC and tested on CCLE.

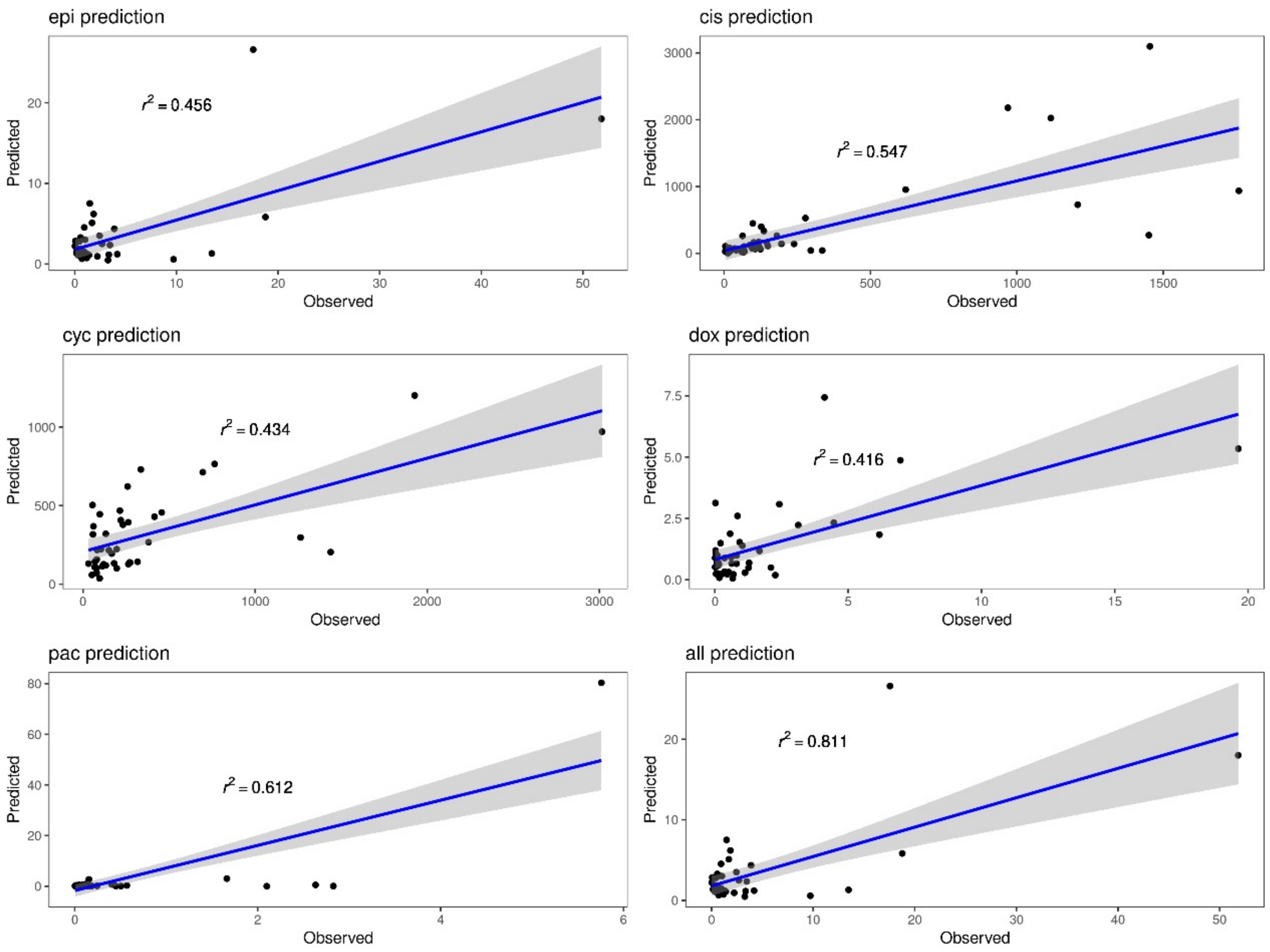

Figure S3. Scatter plots of true and predicted values of five drugs and their combination based on XGBoost-GLM.

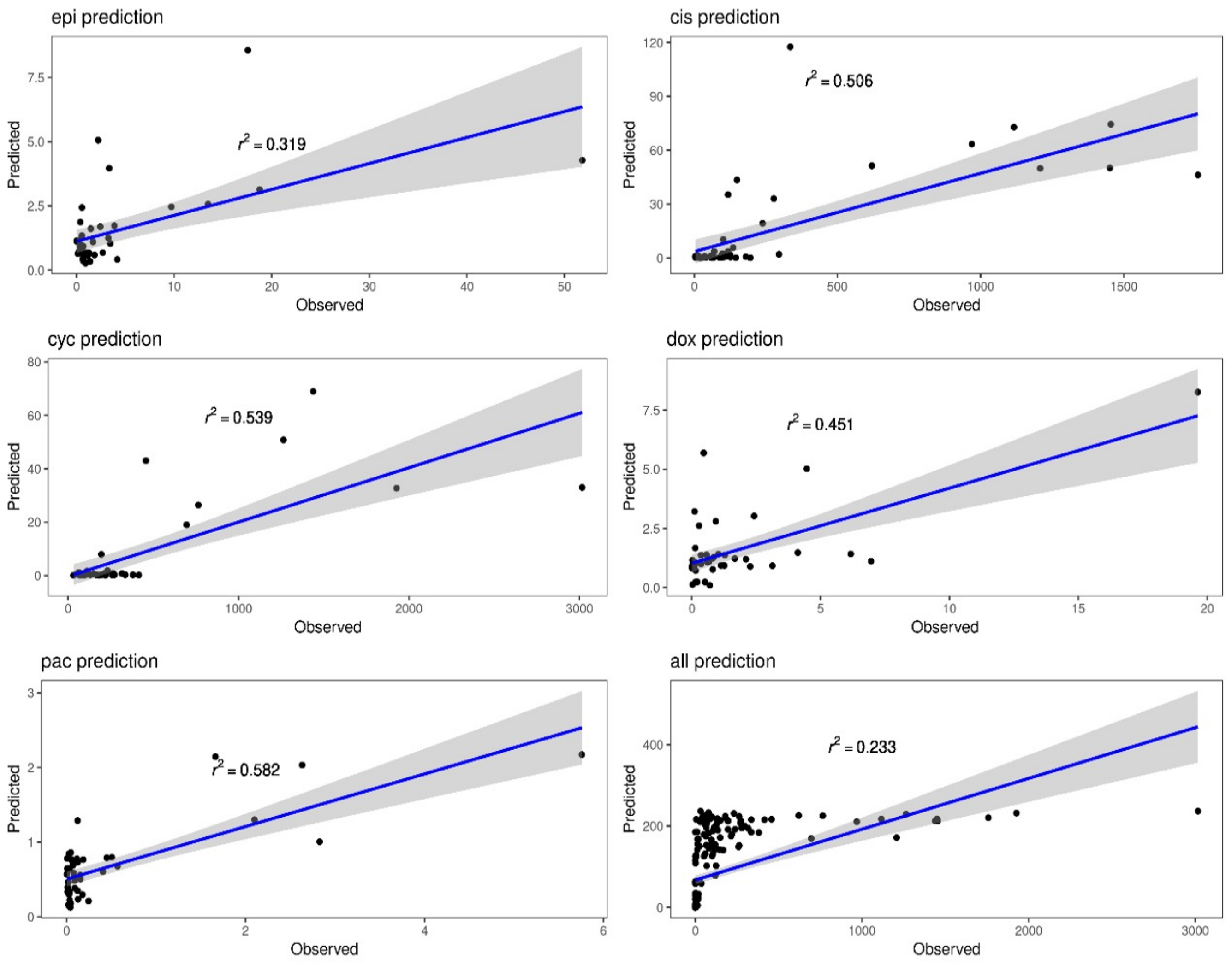

Figure S4. Scatter plots of true and predicted values of five drugs and their combination based on Boruta-mRMR-ANN.

2 TABLES

Table S1. Using CCLE as the training set and GDSC as the test set, then using GDSC as the training set and CCLE as the test set.

Training set	Test set	Drug	R^2	RMSE	PICP
CCLE	GDSC	Epirubicin	0.730	2.175	0.833
		Cisplatin	0.566	1.352	0.920
		Cyclophosphamide	0.643	0.787	0.890
		Doxorubicin	0.765	2.247	0.832
		Paclitaxel	0.707	1.640	0.833
		All drugs	0.744	0.759	0.940
GDSC	CCLE	Epirubicin	0.693	1.454	0.902
		Cisplatin	0.548	1.073	0.804
		Cyclophosphamide	0.749	1.248	0.881
		Doxorubicin	0.766	2.705	0.830
		Paclitaxel	0.638	1.902	0.848
		All drugs	0.780	0.700	0.950

Table S2. The results of XGBoost-GLM and Boruta-mRMR-ANN.

Model	Drug	R^2	RMSE	PICP
XGBoost-GLM	Epirubicin	0.456	1.639	0.902
	Cisplatin	0.547	1.554	0.804
	Cyclophosphamide	0.434	0.888	0.857
	Doxorubicin	0.416	1.881	0.800
	Paclitaxel	0.612	1.952	0.844
	All drugs	0.811	0.648	0.950
Boruta-mRMR-ANN	Epirubicin	0.319	1.407	0.951
	Cisplatin	0.506	4.140	0.483
	Cyclophosphamide	0.539	5.533	0.658
	Doxorubicin	0.451	1.953	0.978
	Paclitaxel	0.582	2.284	0.674
	All drugs	0.233	2.296	0.986

Table S3. Number of features retained after each step of feature selections

Method	Drug	RNA	CNV	MUT	Morgan
Input	Epirubicin	16383	16383	16383	0
	Cisplatin	16383	16383	16383	0
	Cyclophosphamide	16383	16383	16383	0
	Doxorubicin	16383	16383	16383	0
	Paclitaxel	16383	16383	16383	0
Boruta	Epirubicin	9574	462	13520	0
	Cisplatin	9735	491	13325	0
	Cyclophosphamide	9422	449	13482	0
	Doxorubicin	9650	472	13354	0
	Paclitaxel	9716	484	13331	0
mRMR	Epirubicin	257	13	230	0
	Cisplatin	316	45	139	0
	Cyclophosphamide	256	18	226	0
	Doxorubicin	143	13	344	0
	Paclitaxel	313	28	161	0
	All drugs	1182	102	830	256
XGBoost	Epirubicin	16	0	13	0
	Cisplatin	19	2	7	0
	Cyclophosphamide	6	15	12	0
	Doxorubicin	17	1	14	0
	Paclitaxel	10	1	16	0
	All drugs	15	5	0	10
